# Stem cell quiescence: the challenging path to activation

**DOI:** 10.1242/dev.165084

**Published:** 2021-02-08

**Authors:** Noelia Urbán, Tom H. Cheung

**Affiliations:** 1 Institute of Molecular Biotechnology of the Austrian Academy of Sciences (IMBA), Vienna Biocenter Campus (VBC), Dr. Bohr Gasse 3, 1030 Vienna, Austria; 2 Division of Life Science, Center for Stem Cell Research, Center of Systems Biology and Human Health, State Key Laboratory of Molecular Neuroscience, and Molecular Neuroscience Center, Hong Kong University of Science and Technology, Clear Water Bay, Kowloon, Hong Kong, China; 3 Hong Kong Center for Neurodegenerative Diseases, Hong Kong Science Park, Hong Kong, China; 4 Guangdong Provincial Key Laboratory of Brain Science, Disease and Drug Development, The Hong Kong University of Science and Technology Shenzhen Research Institute, Shenzhen-Hong Kong Institute of Brain Science, Shenzhen, Guangdong 518057, China

**Keywords:** Developmental biology, Cell cycle, Stem cells

## Abstract

Quiescence is a cellular state in which a cell remains out of the cell cycle but retains the capacity to divide. The unique ability of adult stem cells to maintain quiescence is crucial for life-long tissue homeostasis and regenerative capacity. Quiescence has long been viewed as an inactive state but recent studies have shown that it is in fact an actively regulated process and that adult stem cells are highly reactive to extrinsic stimuli. This has fuelled hopes of boosting the reactivation potential of adult stem cells to improve tissue function during ageing. In this Review, we provide a perspective of the quiescent state and discuss how quiescent adult stem cells transition into the cell cycle. We also discuss current challenges in the field, highlighting recent technical advances that could help overcome some of these challenges.

## Abstract (precis)

**Summary:** This Review provides a perspective of the quiescent state of adult stem cells and discusses how recent technical advances could help overcome some of the challenges in the field.

## Introduction

Tissue-specific stem cells, also known as adult stem cells, reside in somatic adult tissues, where they contribute to tissue homeostasis and repair (Li and Clevers, 2010). Unlike typical somatic cells, adult stem cells do not contribute to normal tissue function but rather serve as a reservoir of cells that can give rise to multiple highly specialized cell types. During ageing or disease, the number and/or activity of adult stem cells declines, hindering the replacement of aged (or malfunctioning) cells and therefore contributing to the declining performance of tissues (Goodell and Rando, 2015; Schultz and Sinclair, 2016; Tümpel and Rudolph, 2019). This decline in stem cell function is due to both intrinsic mechanisms and changes in extrinsic signals from the environment.

Adult stem cells in different tissues use various strategies to ensure their maintenance over the lifespan of the organism (Mohammad et al., 2019). One such strategy is to remain in a non-proliferative state, called quiescence, which is thought to protect the DNA of cells from mutations acquired during successive rounds of cell division (Walter et al., 2015). Indeed, adult stem cells that exist in a quiescent state can be found in many tissues, but the proportion of quiescent adult stem cells appears to be more prevalent in low turnover tissues, such as skeletal muscle or brain, when compared with rapidly renewing tissues, such as the skin or the gut (Clevers and Watt, 2018). The blood is one high turnover ‘tissue’ that is exempt from this generalization; hematopoietic stem cells (HSCs) remain mostly quiescent and give rise to multipotent progenitors to sustain blood cell production during normal homeostasis (Crane et al., 2017; Nakamura-Ishizu et al., 2014; Pinho and Frenette, 2019). Quiescence is essential for the long-term maintenance of adult stem cells and tissue functions. Excessive quiescence can lead to the generation of too few proliferative cells to cope with the homeostatic needs of tissues (Cheung and Rando, 2013). In contrast, insufficient quiescence can lead to the formation of tumours or, when stem cell activation is not linked to self-renewal, to exhaustion of the stem cell pool. Quiescent stem cells also exist in numerous cancer types, and they contribute to the ability of tumours to evade radiotherapy and chemotherapy (Box 1).

**Box 1. Cancer stem cell quiescence**

Quiescent adult stem cells resist environmental or radiation-induced stress and can regenerate their whole lineage after an insult (Der Vartanian et al., 2019; Doetsch et al., 1999; Scaramozza et al., 2019). Similarly, tumours often efficiently regenerate their full heterogeneity after radiotherapy or chemotherapy, which targets mostly proliferating cells. This observation prompted the hypothesis of the existence of cancer stem cells (CSCs) and suggested that quiescence contributes to the ability of tumours to relapse after treatment (Batlle and Clevers, 2017; Nassar and Blanpain, 2016; Schillert et al., 2013; Sutherland and Visvader, 2015). In line with this, it was noted that disseminated tumour cells that acquired a dormant state are responsible for metastases that appear many years after primary tumour treatment (Giancotti, 2013; Linde et al., 2016; Sosa et al., 2014). Therapies targeting quiescent adult stem cells and niche elements are therefore currently being developed and tested. However, this approach faces similar challenges to studies of adult stem cell biology, namely significant heterogeneity between tumours and within the tumour, and a lack of unique markers of quiescence or dormancy to allow for targeted therapies. An additional challenge for the efficient elimination of CSCs is the enhanced plasticity of CSCs when compared with adult stem cells. This is because some of the differentiated tumour cells regain the ability to generate new CSCs upon CSC ablation (Batlle and Clevers, 2017). In addition, it has been demonstrated that quiescent cells acquire a low immunogenic profile, and that this profile can help cancer stem cells evade the immune system (Agudo et al., 2018; Miao et al., 2019). A special relationship between immune cells and quiescent adult stem cells is also emerging (Naik et al., 2018) but understanding how this relationship is affected in cancer requires further investigation.

Although quiescence has long been considered an inactive state, recent studies have shown that it is actively regulated and modulated by both cell intrinsic and extrinsic signals. Moreover, thanks to technical advances, some studies are beginning to shed light on how quiescent adult stem cells become activated at the molecular level. These include studies of quiescent HSCs, neural stem cells (NSCs) and muscle stem cells (MuSCs), to name a few. In this Review, we highlight some of the general principles governing the maintenance of adult stem cell quiescence and the transition of adult stem cells from the quiescent to the active state. We focus on the main challenges in studying stem cell quiescence and provide an overview of how techniques such as single-cell transcriptomics, proteomic profiling and intravital imaging are advancing our understanding of the quiescent cell state. For a more thorough review of stem cell quiescence in different tissues as well as the mechanisms controlling quiescence, we refer readers to excellent recent reviews (Cho et al., 2019; Mohammad et al., 2019; Naik et al., 2018; Otsuki and Brand, 2020; So and Cheung, 2018; Urbán et al., 2019; van Velthoven and Rando, 2019; Yi, 2017).

## An overview of the reversible quiescent state

**Fig. 1.**
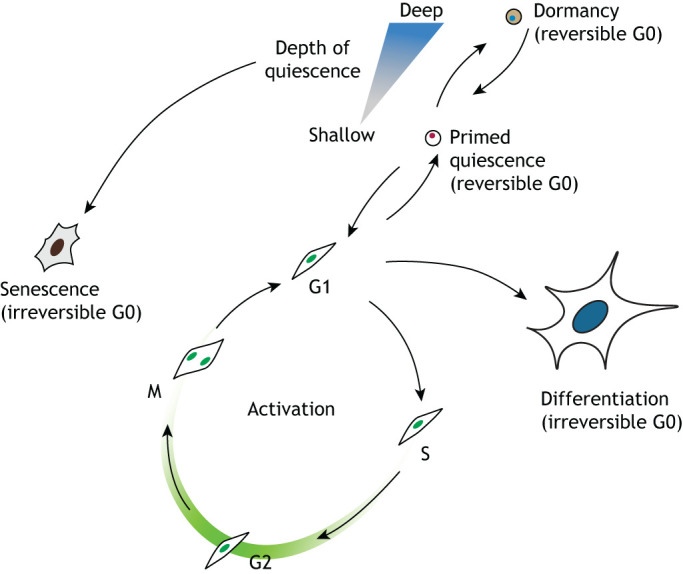
**Cellular states and transitions of adult stem cells.** Quiescent adult stem cells can reversibly transition into an active state in which they enter the cell cycle and generate new differentiated cells to maintain tissue homeostasis. In most tissues, stem cells exist in different depths of quiescence (e.g. the deeply quiescent state called dormancy, or a more shallow quiescent state that is primed for activation). During ageing, or when prompted by pathological states, quiescent stem cells can transition into an irreversible senescent (G0) state, therefore hampering the regenerative potential of the tissue.

An overarching characteristic of all quiescent adult stem cells is that they exist in a reversible G0 cell cycle state. This distinguishes them from differentiated and senescent cells, which exist in an irreversible G0 cell cycle state (Fig. 1). Quiescent cells also display a number of distinct features as well as a certain degree of plasticity. Below, we discuss these features and review how cells enter quiescence, how the quiescent state is maintained and how cells then exit quiescence following activation.

### General features of the quiescent state

Adult stem cells can adopt different quiescent states that differ in their responsiveness to activation signals. One of the best examples is the hematopoietic lineage, where two distinct populations of quiescent HSCs have been identified. Long-term (LT)-HSCs or dormant HSCs have the most significant regenerative potential but they are in a deep state of quiescence and divide only about five times during the lifetime of a mouse (Bernitz et al., 2016; Takizawa et al., 2011; Wilson et al., 2008). LT-HSCs can gradually be activated to become short-term (ST)-HSCs, also called primed or self-renewing HSCs, which are still quiescent but primed to enter the cell cycle (Cabezas-Wallscheid et al., 2017; Wilson et al., 2008). ST-HSCs divide relatively frequently, with a turnover of weeks (Wilson et al., 2008). LT- and ST-HSCs reside in different niche locations and contribute differently to the generation of hematopoietic cells, with ST-HSCs being the main drivers of blood homeostasis and LT-HSCs becoming activated in situations of hematopoietic stress (Szade et al., 2018; Wilson et al., 2007). The distinction between dormant and primed adult stem cells is not so clear for other tissues but, in general, a lighter quiescent, primed or resting state is defined as a temporary inactive phase, while dormancy refers to a deeper and longer state of quiescence. In some cases, differentiated cells retain high levels of plasticity, making it possible to modulate their fate. This is the case for parenchymal astrocytes in the brain, which can be repurposed to generate neurons *in situ* with or without injury stimulation (Qian et al., 2020; Torper and Götz, 2017; Zamboni et al., 2020). Indeed, a recent report used single-cell sequencing to reveal that astrocytes function as deeply dormant NSCs, recapitulating the adult neurogenic lineage upon loss of Notch signalling (Zamboni et al., 2020). As we discuss later, such advances in lineage tracing and single-cell sequencing are beginning to help unearth previously unknown quiescent populations, the relationship between adult stem cells in different depths of quiescence in several tissues, and the relative contributions of different quiescent cells to homeostasis and repair.

Quiescence is also usually associated with an inactive cellular state. For example, cellular quiescence is characterized by a lack of expression of cell cycle-related genes and a global downregulation of mRNA production and protein synthesis, as observed in plants, yeast or even some proliferative mammalian cells, such as fibroblasts that become quiescent in response to serum starvation or contact inhibition. However, the quiescent state of adult stem cells is not just an inactive phase but is actively regulated (Cheung and Rando, 2013). Quiescent adult stem cells in different tissues also share many characteristics beyond the lack of cell cycle markers, such as a close association with their respective niches and the acquisition of metabolic and transcriptional signatures that are different from those of the rest of the tissue (Cho et al., 2019). However, despite the common general features of quiescent adult stem cells, their metabolic and transcriptional signatures in different tissues vary, and a common transcriptional profile of quiescent adult stem cells – or a universal marker – does not exist at the molecular level (Keyes and Fuchs, 2018).

**Fig. 2.**
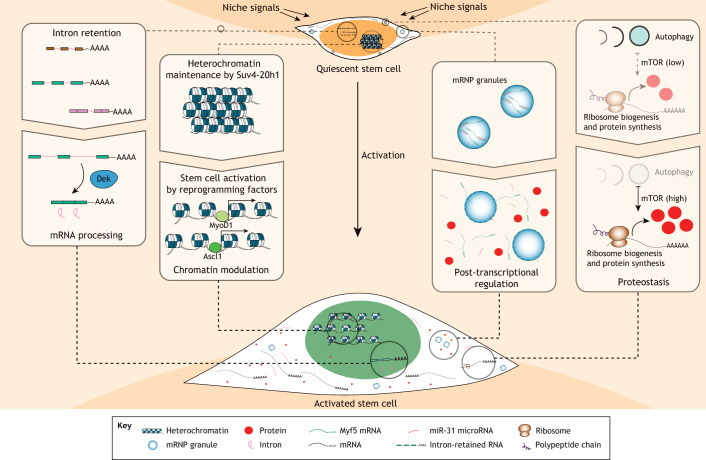
**The features and molecular regulation of adult stem cell quiescence and activation.** Some of the processes that contribute to the activation of quiescent stem cells are depicted. Intron-retained transcripts are accumulated in quiescent stem cells and are processed upon stem cell activation. Important changes in chromatin accessibility have also been reported between quiescent and active stem cells. In addition, key stem cell activation factors, such as MyoD1 and Ascl1, are potent reprogramming factors that harbour the ability to open closed chromatin. Post-transcriptional regulation of gene expression also plays an important role in the quiescent-to-activation transition; this is a process that could be facilitated and/or controlled by phase-separation mechanisms. Finally, protein homeostasis (proteostasis) is emerging as an important regulator of adult stem cells, not only controlling energy metabolism but also the abundance of proteins that act as regulators of the quiescence-to-activation transition.

Adult stem cells are actively regulated, both as they transition into and out of quiescence, and while they are maintained within the quiescent state (Fig. 2). A combination of intrinsic and extrinsic mechanisms controls the quiescent state, including cell cycle and transcriptional regulators, metabolic cues, contact with the extracellular matrix (ECM), and local and systemic cues (Urbán et al., 2019). These signals often exert very different effects on various types of adult stem cells, which reflects the different regulatory needs of the tissues they occupy. Although some tissues are in constant regeneration (e.g. the gut), others hardly ever self-renew (e.g. skeletal muscle) or go through alternating regenerative and pausing phases (e.g. the hair follicle) (Li and Clevers, 2010).

### Entry into the quiescent state

Most of our current knowledge on the mechanisms controlling the entry into quiescence comes from experiments aimed at studying cell cycle progression in yeast or cultured cell lines. Although a detailed analysis of cell cycle progression in adult stem cells is still missing, many of the findings from these *in vitro* systems can be translated into adult stem cell biology. Global levels of activated cyclin-dependent kinases (CDKs) are the primary driver of cell cycle progression. CDK activity is determined by the balance of CDKs, cyclins and CDK inhibitors present within the cell, and is regulated by extrinsic mitogenic signals (Coudreuse and Nurse, 2010; Swaffer et al., 2016). CDK activation levels continuously increase from G1 to M phase, then drop dramatically after mitosis. Certain threshold levels of CDK activity mark a point of no return, termed the ‘restriction’ or ‘R’ point, at which cells commit to entering the S phase (Schwarz et al., 2018). Using a CDK activity reporter in a human breast epithelial cell line, it has been shown that a population of cells quickly recovers CDK activity (and is considered to be in G1), whereas another population maintains low CDK activity levels, resembling a G0 state, for a variable period (Spencer et al., 2013). Furthermore, although the cells in G1 re-enter S phase regardless of their environment, cells with low CDK activity levels require mitogenic signals to progress through the cell cycle. The decision to enter quiescence therefore depends on the niche signals received by daughter cells after division. This provides a mechanism for daughter cells to enter quiescence asymmetrically, independent of the mode of cell division. This has already been suggested to occur in some adult stem cell niches (Simons and Clevers, 2011). The likelihood of a cell re-entering the cell cycle is also influenced by the signalling environment of the mother cell, with proliferating cells that have received high levels of mitogenic signals being more likely to generate daughter cells that re-enter the cell cycle (Yang et al., 2017a). This potential positive feedback loop of proliferation remains to be confirmed in adult stem cell populations.

Cyclins, CDKs and CDK inhibitors have all been shown to play prominent roles in regulating the quiescent state in different tissues (Cho et al., 2019). In particular, the CDK inhibitors p21, p27 and p57 (CDKN1A, CDKN1B and CDKN1C, respectively) are crucial for the quiescence of multiple adult stem cell populations, including hair follicle stem cells (HFSCs), HSCs and NSCs (Andreu et al., 2015; Cheng, 2000; Furutachi et al., 2013; Lee et al., 2013; Matsumoto et al., 2011; Porlan et al., 2013). The exact time at which adult stem cells enter quiescence has not been defined for most tissues. In the subventricular zone niche in the brain, adult NSCs enter quiescence at mid-embryonic stages, which is much earlier than previously thought (Fuentealba et al., 2015; Furutachi et al., 2015). The early entry of NSCs into quiescence depends on p57 expression (Furutachi et al., 2015) and is thought to preserve the neurogenic potential of NSCs before they become gliogenic later in development.

It is commonly assumed that cells enter quiescence immediately after mitosis, sometime in the early G1 phase of the cell cycle before reaching the R point. This has been recently challenged by the finding that a fraction of *Drosophila* larval NSCs (termed neuroblasts) enter quiescence while in G2 (Otsuki and Brand, 2018). These G2-arrested neuroblasts progress faster through the cell cycle once re-stimulated, providing a mechanism that introduces heterogeneity into the response of the cell to external signals. However, the existence of G2-arrested adult stem cells in mammals remains to be confirmed. One challenge when trying to determine the exact time at which a cell has entered G0 is that the transition from proliferation to quiescence remains poorly defined, with most markers showing a graded pattern of expression. The degree of accumulation of certain cell cycle-related proteins has thus been used as a proxy for quiescence entry. Expression of the classical cell cycle marker Ki-67, for example, gradually increases from S phase and peaks at mitosis. As the protein is steadily degraded during the G0 and G1 phases, the levels of Ki-67 can be used to infer how long a cell has been in G0/G1 (Miller et al., 2018). The accumulation of a defective form of the cell cycle inhibitor p27 has also been used to distinguish G0 (high levels) from G1 (low-mid levels), and even to identify quiescent MuSCs *in vivo* (Oki et al., 2014).

### Maintaining the quiescent state

A number of mechanisms have been shown to contribute to maintaining the quiescent state. For example, quiescent human somatic cells actively replenish CENP-A nucleosomes to preserve centromere identity and proliferative potential (Swartz et al., 2019). Quiescent NSCs maintain high levels of expression of genes involved in signalling to rapidly react to external stimuli (Shin et al., 2015). Cell adhesion as well as several signalling pathways have been linked to the maintenance of quiescence in specific adult stem cell populations, although with high variability between different tissues. Notch signalling, for example, promotes quiescence in NSCs and MuSCs (Bjornson et al., 2012; Mourikis et al., 2012; Sueda and Kageyama, 2020; Zhang et al., 2018), but induces proliferation of HFSCs through an indirect mechanism involving immune cells (Ali et al., 2017).

The maintenance of quiescent and active stem cells also relies on cellular processes such as autophagy or lysosomal function, highlighting the importance of proteostasis for stem cells (Baser et al., 2017; García-Prat et al., 2017). Macroautophagy (often simply referred to as autophagy) allows cells to digest cellular components to obtain energy. Autophagy is active in quiescent adult stem cells and acts to provide a source of macromolecules, regulate metabolism by eliminating mitochondria (mitophagy) and prevent senescence (Boya et al., 2018; Casares-Crespo et al., 2018). The levels of autophagy decrease globally during ageing, which has been linked to the decline in function of adult stem cells in different tissues (García-Prat et al., 2016; Ho et al., 2017; Leeman et al., 2018). Lysosomal degradation is also particularly active in quiescent NSCs, where it is required for the maintenance of quiescence (Kobayashi et al., 2019). A recent study found that lysosomal gene expression is highest in deeply quiescent MuSCs, likely compensating for a reduction in autophagy in these cells (Fujimaki et al., 2019). Stimulating lysosomal function pushes the cells towards activation while inhibiting it induces a senescent signature. This suggests that the levels of lysosomal activity work as a dimmer switch for a continuum of cellular states, from shallow quiescence to deep quiescence and finally to senescence (Fujimaki et al., 2019). The seemingly contradictory effects of modulating autophagy or lysosomal function reflect the complexity of the regulatory network governing the quiescent state and warrant further investigation. Although proteostasis is emerging as a master regulator of quiescence, so far quiescence studies have heavily relied on transcriptional data and, therefore, might be missing some of the regulatory mechanisms that control this state.

The niche, as well as external stimuli, also plays a crucial role in regulating the maintenance and depth of stem cell quiescence. For example, muscle fibres secrete Wnt4, which maintains the quiescent state of MuSCs by restricting their mobility and by controlling YAP activation through a RhoA-dependent mechanism (Eliazer et al., 2019). Stimuli such as injuries, even when in a distant organ, can also push different types of quiescent adult stem cells to a shallower quiescent state, referred to as G-alert, in which they can rapidly respond to further insults (Rodgers et al., 2014). This primed state depends on the activity of the master metabolic regulator mammalian target of rapamycin (mTOR), which stimulates protein synthesis and inhibits autophagy (Fig. 2) (Meng et al., 2018; Rafalski and Brunet, 2011; Rodgers et al., 2014).

### Exit from the quiescent state

Exit from the quiescent state is characterized by the graded accumulation or depletion of key regulators. For example, CDK6 levels gradually increase during the exit of HSCs from quiescence (Laurenti et al., 2015). By contrast, the level of the microRNA mir-489, which is highly expressed in quiescent MuSCs, steadily decreases upon activation (Cheung et al., 2012). One of its direct targets, Dek, is expressed in most proliferating cells and regulates mRNA by facilitating splicing of intron-retained transcripts that accumulate in quiescent MuSCs (Cheung et al., 2012; Yue et al., 2020). Interestingly, the phenomenon of intron retention appears to be conserved and can be found in different types of quiescent adult stem cells (Yue et al., 2020). Thus, the readiness of a cell to re-enter the cell cycle depends on the balance of intrinsic pro-activating and pro-quiescence factors at any given time.

Exit from quiescence can be triggered by a number of different factors. Tissue disruption, e.g. upon injury, can induce the activation of adult stem cells and does so via diverse mechanisms. These include the loss of cell-cell contacts and cell-ECM contacts that maintain quiescence. In addition, the release of stimulating factors that are captured by the ECM or present in circulating blood can also activate adult stem cells (Cho et al., 2019). Signals coming from the immune system also induce the proliferation of quiescent adult stem cells. Although chronic interferon A (IFNA) is detrimental to HSCs, a short pulse can activate dormant LT-HSCs, promoting blood regeneration (Essers et al., 2009). In skeletal muscle, the genetic disease Duchenne Muscular Dystrophy (DMD) is characterized by progressive muscle degeneration due to a mutation in the dystrophin gene. Dystrophin-deficient muscle fibres are not able to maintain muscle integrity. As a result, the muscle regenerates repeatedly and triggers the reactivation of MuSCs, eventually depleting the stem cell pool (Guiraud et al., 2015; Nowak and Davies, 2004). In the brain, pathological stimuli such as epileptic seizures can also activate quiescent NSCs. Intense seizures cause the conversion of NSCs into reactive astrocytes, but milder epileptogenic activity is enough to activate previously quiescent adult NSCs (Pineda and Encinas, 2016; Sierra et al., 2015).

## Recent advances and current challenges for the study of stem cell quiescence

Modulating the quiescent state of adult stem cells and triggering stem cell activation could be a powerful way of controlling tissue regeneration and counteracting ageing (Goodell and Rando, 2015; Mahmoudi et al., 2019). However, quiescent adult stem cells have proved to be challenging to access, and long-term treatments based on their manipulation are still far from being a reality. Below, we discuss the main roadblocks that have hampered studies of quiescent adult stem cells and highlight recent discoveries that, we feel, will allow significant advances in the field.

### The prospective isolation of quiescent stem cells

To study and characterize quiescent adult stem cells, it is useful to have an approach that allows for the isolation and purification of these cells. To date, however, this has remained a challenge. Stem cell identity is generally defined by behaviour or function (Clevers and Watt, 2018), in other words, the ability of cells to self-renew and differentiate into different cell types (McCulloch and Till, 2005). Although stem cell isolation largely relies on the presence of specific markers, no universal markers of adult stem cells exist, and tissue-specific stem cells are generally identified by the expression of different sets of characteristic genes. For example, Lgr5 is commonly used to identify intestinal stem cells (Barker et al., 2007), whereas Pax7 is used for MuSCs (Seale et al., 2000), and Sox2 and nestin are used for NSCs (Ellis et al., 2004; Lagace et al., 2007). In some cases, a combination of markers is needed due to the lack of exclusive stem cell markers (e.g. Sox2 marks NSCs and astrocytes, whereas nestin labels NSCs and more committed progenitors). One immediate consequence of the lack of universal markers of stem cells, even in the same tissue, is that the use of advanced genetic approaches can result in different interpretations depending on the mouse line used for genetic lineage tracing. In the adult brain, nestin- and glast-labelled stem cells are mostly quiescent and have long-term neurogenic potential, whereas Ascl1-positive stem cells divide frequently and have mainly short-term neurogenic potential (Kim et al., 2007; Mich et al., 2014; Pilz et al., 2018). Another caveat of lineage tracing approaches using Cre-ERT2 lines is the dose of tamoxifen, which can not only generate an injury-like environment, as reported in the stomach (Keeley et al., 2019), but can also select for small subsets of stem cells that might not be representative of the overall population (Bonaguidi et al., 2011; Rios et al., 2016). All of these concerns are exacerbated by age and certain pathological conditions, which can alter the expression of these markers. Moreover, and as highlighted above, there is no universal marker for quiescence, thus the quiescent state tends to be defined by a combination of molecular features (Fig. 2). For example, quiescent cells are characterized by their low RNA and protein content, and their cell cycle state. Indeed, the absence of cell cycle proteins such as Ki67 is often used in conjunction with tissue-specific stem cell markers to define quiescent adult stem cell populations.

Fortunately, with the increasing high-throughput capacity of single cell RNA-sequencing (scRNA-seq), it is no longer necessary to sort or identify cells prior to sequencing, and bioinformatic tools now allow the identification of cell types from whole-tissue sequencing (Hochgerner et al., 2018). Although cluster identification still relies on the presence of characteristic marker genes, this approach is much less biased than sorting stem cells based on the expression of a small subset of markers. Moving forward, the data generated from this approach could lead to the identification of better markers or combinations of markers for adult stem cells and quiescence in different tissues (Dulken et al., 2017; Giordani et al., 2019). This will better help identify the changes occurring during the transition of stem cells between active and quiescent states by eliminating confounding effects due to contaminating cell populations during cell isolation or identification.

### Tackling stem cell heterogeneity

In the past few years, scRNA-seq studies have clearly shown that the stem cell state is not distinct but rather a continuous transition from one state to another. This applies to the transition of a stem cell to more differentiated progeny and also to the transition from deep quiescence to active states (Cabezas-Wallscheid et al., 2017; Dulken et al., 2017; Giordani et al., 2019; Hochgerner et al., 2018; Yang et al., 2017b). These scRNA-seq studies have also confirmed that much more than the cell cycle is regulated during the exit from quiescence. Activated NSCs and MuSCs, for example, have higher overall rates of gene expression and protein synthesis than their quiescent counterparts (Shin et al., 2015; Machado et al., 2017; van Velthoven et al., 2017). Metabolic changes can in fact act as drivers of the transition of stem cells between quiescent and active states, as demonstrated in NSCs, where blocking fatty acid oxidation (FAO) leads to their activation (Knobloch et al., 2017). It has been shown that quiescent adult stem cells have distinct metabolic characteristics, which vary significantly between niches. In the bone marrow, quiescent HSCs use glycolysis and FAO for energy production and increase the use of the mitochondrial respiratory chain in the active state (Nakamura-Ishizu et al., 2020). In contrast, in the muscle, quiescent MuSCs preferentially use FAO, switching to glycolysis as a primary source of ATP upon activation (Ryall et al., 2015). Studies using scRNA-seq have also helped to identify metabolic changes in other adult stem cell niches, such as NSCs, which transition from using aerobic glycolysis and FAO in quiescence to using oxidative phosphorylation by mitochondria and increasing lipogenesis when activated (Llorens-Bobadilla et al., 2015). A recent scRNA-seq study of skeletal muscle described the cell states corresponding to different stages along this transition, as well as their metabolic signatures (Dell'Orso et al., 2019).

In many cases, scRNA-seq has revealed unexpected heterogeneity in populations of adult stem cells that were previously thought to be homogeneous. The intestinal epithelium is a high turnover tissue maintained by highly proliferative Lgr5-positive intestinal stem cells. However, scRNA-seq studies have identified a new, slowly cycling sub-population of Lgr5-positive stem cells characterized by the expression of the RNA binding protein Mex3a (Barriga et al., 2017). These Mex3a-/Lgr5-positive stem cells survive chemotherapy and radiation, and mediate regeneration after these toxic insults, and were thus proposed to be in a state of quiescence as both chemotherapy and radiation target mostly cycling cells (Barriga et al., 2017). Similarly, scRNA-seq revealed heterogeneity among quiescent HFSCs, with HFSCs being differentially primed for diverse lineages depending on their exact location within the niche (Yang et al., 2017b). Moreover, in most tissues, stem cells do not activate synchronously and, accordingly, scRNA-seq studies have unearthed heterogeneity of adult stem cells along the quiescence-to-activation trajectory, e.g. in the case of MuSCs in skeletal muscle and NSCs in the ventricular-subventricular zone of the brain (Dell'Orso et al., 2019; Llorens-Bobadilla et al., 2015).

### Characterizing epigenetic modifications

Epigenetic mechanisms are tightly linked to ageing and metabolism, and play an essential role in adult stem cell maintenance (Beerman and Rossi, 2015; Brunet and Rando, 2017; Ren et al., 2017). As such, epigenetic changes might be partly responsible for the profound transcriptional changes adult stem cells undergo when exiting the quiescent state. There is already evidence to suggest that chromatin changes extensively during the activation of quiescent adult stem cells in some tissues (Boonsanay et al., 2016). For example, in early activated MuSCs, regions of chromatin that are marked by H3K4me3, which is indicative of a permissive chromatin environment for active transcription (Bernstein et al., 2002; Santos-Rosa et al., 2002), and by H3K27ac, which has been associated with active enhancers (Creyghton et al., 2010), increase significantly compared with quiescent MuSCs (Machado et al., 2017). Indeed, during ageing, quiescent MuSCs and HSCs are more difficult to activate and display increased levels of repressive epigenetic marks such as H3K27me3 (Liu et al., 2013; Sun et al., 2014).

However, epigenetic modifications remain challenging to study in quiescent adult stem cells, mostly owing to difficulties in obtaining enough material to perform the necessary molecular assays. Significant advances have been made in recent years to minimize the cell input required for techniques such as ChIP-seq (Schmidl et al., 2015), Hi-C (Lieberman-Aiden et al., 2009; Nagano et al., 2013) and ATAC-seq (Buenrostro et al., 2013). In some of these cases, the techniques can be scaled down to single cell resolution (Buenrostro et al., 2015; Nagano et al., 2013). Such innovations, together with genetic approaches, are already yielding unprecedented information on the chromatin states of adult stem cells in quiescence and upon activation. For example, it has been demonstrated that the H4K20 dimethyltransferase Suv4-20h1 is essential for MuSC quiescence, where it acts by reducing chromatin accessibility and maintaining heterochromatin formation (Boonsanay et al., 2016). In addition, imaging of the cell polarity regulator Cdc42 (to follow the fate of daughter cells), followed by the use of single cell ATAC-seq in combination with scRNA-seq, revealed that epigenetic asymmetry of HSCs after cell division is linked to the retention of stem cell potential (Florian et al., 2018).

Many of the crucial activation factors for adult stem cells, such as MyoD1 for MuSCs or Ascl1 for NSCs (Andersen et al., 2014; Wang et al., 2014), are also potent reprogramming factors that harbour the ability to change the chromatin landscape of cells (Davis et al., 1987; Treutlein et al., 2016). In the adult hippocampal niche of the brain, quiescent NSCs that have been proliferating (and therefore express high levels of Ascl1 protein) behave differently to NSCs that remain quiescent, as they are much more likely to become activated again (Urbán et al., 2016). This opens up the possibility that quiescent NSCs do not fully recover their previous chromatin state after being activated, which might confer further heterogeneity to the stem cell pool. Chromatin conformational changes might also underlie the higher activation potential of primed stem cells in response to injury, although this also remains to be addressed. Such analysis is now possible with the advancement of techniques such as ATAC-seq or HiC analysis.

### Analysing proteomic changes at single cell resolution

Recent advances in transcriptomics, although remarkable, are not enough to provide a complete picture of the quiescent state and its regulation. This is because critical modulators are often highly unstable proteins that show poor correlation between protein and mRNA levels (Blomfield et al., 2019; Boutet et al., 2012; Cheung et al., 2012; Crist et al., 2012). Post-transcriptional and post-translational regulation plays an essential role in maintaining stem cell quiescence. For example, pro-proliferation factors are prevented from being expressed in the quiescent state by microRNAs (Cheung et al., 2012; Crist et al., 2012). In many cases, the proteins of proliferation factors are absent in quiescent adult stem cells, while their transcripts are abundantly expressed to achieve a quiescent state that can be quickly activated upon signal modulation (Blomfield et al., 2019; Cheung et al., 2012; Crist et al., 2012). Post-translational modifications are also crucial to determine the function of most effector proteins, particularly during cellular transitions such as stem cell activation from quiescence. As such, detailed molecular characterization of quiescence exit or re-entry at the protein level is required.

While advances in single-cell proteomics have been – and continue to be – made, the application of these techniques to study stem cell quiescence is in its infancy. Single cell mass cytometry time of flight (CyTOF) is an approach used to capture the temporal dynamics of transcription factor (TF) expression in individual cells (Palii et al., 2019). This technique has revealed that quantitative changes in protein abundance of lineage-specific TFs in progenitors during human erythropoiesis can determine alternate cell fates (Palii et al., 2019). Similar approaches have also been applied to study the diversity of tissue-resident cells in skeletal muscle, which have previously been characterized solely based on scRNA-seq data (Giordani et al., 2019; Porpiglia et al., 2017). In addition to mass spectrometry, new technologies are being developed to identify the cell surface receptors of a cell population at single cell resolution along with scRNA-seq (Shahi et al., 2017; Stoeckius et al., 2017; van Eijl et al., 2018). Cell surface receptors are probed with antibodies tagged with DNA sequence barcodes that can be read out at the single cell level using DNA sequencing, offering the advantages of high sensitivity, accuracy and virtually limitless multiplexing. Although still underdeveloped, these technologies could help reveal the molecular mechanisms underlying stem cell heterogeneity both in homeostasis and during exit from and re-entry to quiescence. Furthermore, they could provide a potential set of cell surface markers for prospective isolation of specific stem cell populations, including quiescent adult stem cells.

### Capturing quiescence *in situ*

Niche signals are crucial for stem cell quiescence. Therefore, it is not surprising that tissue dissociation is sufficient to activate adult stem cells. Indeed, MuSCs dramatically change their expression profile due to the dissociation protocol alone (Machado et al., 2017; van Velthoven et al., 2017). This raises the question of whether the characteristic signatures of primed stem cell clusters identified using scRNA-seq techniques might be, at least in part, due to dissociation artefacts. The importance of the niche goes even further and in extreme cases, such as in the intestine, niche cells (Paneth cells) are required to support Lgr5-positive stem cells metabolically, allowing them to form colonies *in vitro* (Rodríguez-Colman et al., 2017; Sato et al., 2011). Therefore, both the transcriptional signature and the intrinsic potential of adult stem cells are altered when they are detached from the niche. In recent years, several new approaches have been developed to help overcome this problem by allowing the study of stem cells in their native state.

One such approach involves fixing stem cells *in situ* as soon as possible after dissection to preserve their *in vivo* signatures. The use of RNA synthesis inhibitors during the isolation of MuSCs also prevents changes in transcription that are induced by the removal of stem cells from their niche (van Velthoven et al., 2017). Fixation *ex vivo* immediately after tissue isolation or systemically through perfusion of fixatives can also help capture the authentic quiescent signature of adult stem cells (Machado et al., 2017; van Velthoven et al., 2017; Yue et al., 2020). One of the caveats of these methods is that the RNA isolated from fixed samples is densely crosslinked and of low quality. Recently, perfusion using light fixative has been shown to overcome this issue, significantly improving the quality of the isolated RNA (Yue et al., 2020). Interestingly, this technique revealed that Notch signalling downstream targets, such as Hes1 and Heyl, are detected in fixed quiescent MuSCs but not in freshly isolated MuSCs (i.e. not fixed). Thus, this approach appears to be able to capture the real transcriptional signature of adult stem cells, including readouts of their interactions with the niche. By varying the time of fixation after isolation from the niche while preserving genuine *in situ* molecular signatures, it will be possible to examine the profile of stem cells at defined times after activation, for example during the early windows of stem cell activation that are currently missed during conventional isolation processes. This will potentially unearth the molecular mechanisms that are crucial for exit from quiescence and early activation. Other strategies initially developed to improve RNA-sequencing quality could also help preserve quiescent cell gene expression. For example, freezing fresh tissue followed by the sequencing of single-cell nuclei has been shown to preserve RNA integrity and improve sequencing data quality (Guillaumet-Adkins et al., 2017).

Another technique with great potential to help unravel the real quiescent signature *in vivo* is high throughput *in situ* hybridization (Eng et al., 2019; Lee et al., 2014; Rodriques et al., 2019; Wang et al., 2018). This approach has the added advantage of retaining positional information and can allow a detailed characterization of not only adult stem cells but also the niche cells that regulate them.

### Visualizing quiescent adult stem cells *in vivo*

Certain aspects of stem cell behaviour, such as stem cell dynamics, remain hard to study even with high quality *in situ* expression data for stem cells and their niche. This is particularly evident when trying to understand direct cell-cell interactions or lineage dynamics. In the case of lineage-tracing experiments, reconstructed lineages are usually inferred from a few time points after labelling, which rarely reflect the actual dynamics of stem cell divisions. Imaging-based approaches, such as intra-vital imaging, therefore have the potential to uncover unique features of quiescent and activated adult stem cells, and their interaction with the niche.

A characteristic feature of quiescent adult stem cells arising from live-imaging studies in different tissues is that they have minimal mobility, strengthening the importance of their close association to niche components (Christodoulou et al., 2020; Pilz et al., 2018; Webster et al., 2016). The visualization of HSCs in the bone marrow using multimodal imaging has revealed structural and functional heterogeneity between HSCs from different niche locations (Lassailly et al., 2013). More recently, the live tracking of quiescent HSCs in different bone locations revealed striking differences in their amplification potential that are directly related to the characteristics of their microenvironment (Christodoulou et al., 2020). In another study, multipoint intravital time-lapse confocal microscopy was used to reveal differences between active and quiescent HSCs with regard to their interactions with the niche. This approach showed that, although quiescent HSCs stably interact with a small region of the niche, activated HSCs are motile and have a higher number of interactions with distinct niche locations (Rashidi et al., 2014). In the skeletal muscle compartment, quiescent MuSCs indeed have very low mobility. However, upon injury, they migrate along the damaged myofibres, also called ghost fibres, to the injury site (Webster et al., 2016).

Intra-vital imaging has also revealed the mode of division of adult stem cells, often giving a much more complex picture than previously thought. This is the case in the adult hippocampus, where adult NSCs were shown to divide in a wide array of modes (Pilz et al., 2018). These include self-renewing and depleting symmetric divisions, as well as asymmetric divisions with diverse outcomes, e.g. the direct generation of post-mitotic neurons. In addition, live-imaging of the mammary gland revealed that multipotent stem cells do not exist in this niche; instead, a heterogeneous pool of fate-restricted stem cells together confers multipotency to the tissue (Scheele et al., 2017).

Intra-vital imaging can be a very powerful tool for the study of adult stem cells when combined with genetic tools to disturb their properties. In the hair follicle, for example, single cell tracking and manipulation of stem cells in live mice revealed unexpected flexibility of lineage choice not only after injury but also under homeostatic conditions (Xin et al., 2018). The use of similar approaches in different tissues will hopefully shed more light on how quiescent adult stem cells are activated and how they contribute to homeostasis and to repair after injury.

## Conclusions and future perspectives

Rapidly developing technologies are contributing to our understanding of the quiescent state at unprecedented speed. However, there are still important aspects of quiescence that remain obscure, mostly owing to the lack of proper tools to investigate them *in vivo*, together with the intrinsic scarcity of quiescent adult stem cells. For example, it is still unclear how adult stem cells integrate the signals they receive from the niche to mount an appropriate regenerative/homeostatic response. Some progress has been made identifying crosstalk between the pathways controlling quiescence. An example of this is the dampening of the Akt pathway by ERK signalling that occurs in activated HSCs, allowing them to return to quiescence (Baumgartner et al., 2018). Advances in single-cell proteomics, together with *in situ* imaging and the development of sensitive reporter lines for pathway activation, will undoubtedly help us further unravel such signal integration mechanisms. The potential asymmetric distribution of molecules and cellular compartments between daughter cells is also gaining attention as another determinant for the re-entry into quiescence after division. In addition to the well-known differential distribution of cell cycle determinants, some adult stem cells can differentially segregate damaged proteins between daughter cells. This phenomenon relies on a diffusion barrier in the endoplasmic reticulum at the time of mitosis and has been proposed to preserve the fitness of the cell retaining stem cell properties (Moore et al., 2015). The differential segregation of organelles or even metabolic components/metabolites could also have a significant impact on cell fate decisions, but so far has barely been explored.

As discussed above, quiescent adult stem cells undergo dramatic changes as they enter the cell cycle. Activation is associated with increases in RNA and protein content, relaxation of heterochromatin and extensive changes in metabolism that require a significant reorganization of biological molecules within cellular compartments and organelles (Fig. 2). Profound changes also occur during other cellular transitions, such as during differentiation or entry into senescence during ageing. In the case of quiescence, however, the changes must be reversible to allow stem cell reactivation. Ageing or disease might hamper such reorganization, therefore affecting stem cell transitions and potentially their long-term maintenance and function. Thus, gaining a better understanding of how a quiescent cell orchestrates this reorganization could further our understanding of stem cell functions in ageing or disease.

The process of phase separation, which is involved in the formation of membrane-less organelles (Boeynaems et al., 2018), is also emerging as a regulator of adult stem cell quiescence. In quiescent MuSCs, the microRNA miR-31 and its target Myf5 are sequestered in membrane-less messenger ribonucleoprotein (mRNP) granules. During activation, granule dissociation increases the chance of Myf5 mRNA escaping repression by miR-31, allowing Myf5 protein translation (Crist et al., 2012). Phase separation could, therefore, be functionally crucial to prime quiescent MuSCs for activation in a post-transcriptional manner. Interestingly, cellular ATP can act as a biological hydrotrope that controls phase separation and aggregation (Patel et al., 2017). ATP levels increase significantly during the activation of MuSCs and HSCs, potentially altering the liquidity of membraneless organelles, such as mRNP granules, to orchestrate the fast reorganization of macromolecules needed for the quiescence-to-activation transition. Considering that exit from quiescence involves precise transcriptional, post-transcriptional and epigenetic control to reprogram the cellular state, phase separation could provide a fast and orderly way of reorganizing the transcriptional and translational machinery of stem cells, thereby facilitating, or even promoting, the transition towards a proliferative cell state.

The modulation of adult stem cell quiescence holds great potential to increase tissue repair, both after injury and during normal ageing. Interventions aimed at improving the activation of quiescent adult stem cells have already been proposed as a rejuvenation strategy in some tissues. However, before we can safely apply these interventions, we must investigate the long-term consequences of altering quiescent adult stem cell populations. It will be crucial to determine, on a tissue-by-tissue basis, whether stem cell activation leads to loss of the stem cell population and, therefore, to a further loss of tissue function at later time points. To prevent this, any interventions increasing stem cell activation should be accompanied by the promotion of regulated self-renewal to ensure the long-term maintenance of stem cells and their regenerative potential. Further studies that aim to better understand the quiescent state and how it is regulated will therefore undoubtedly aid the development and application of regenerative and rejuvenating strategies.
